# Reciprocal modulation of Aβ42 aggregation by copper and homocysteine

**DOI:** 10.3389/fnagi.2014.00237

**Published:** 2014-09-08

**Authors:** Salla Keskitalo, Melinda Farkas, Michael Hanenberg, Anita Szodorai, Luka Kulic, Alexander Semmler, Michael Weller, Roger M. Nitsch, Michael Linnebank

**Affiliations:** ^1^Department of Neurology, University Hospital ZurichZurich, Switzerland; ^2^Division of Psychiatry Research, University of ZurichSchlieren, Switzerland

**Keywords:** homocysteine, Alzheimer’s disease, copper, Aβ, cytotoxicity, primary neurons

## Abstract

Hyperhomocysteinemia is a risk factor for Alzheimer’s disease (AD). Both homocysteine (Hcy) and amyloid β (Aβ), which accumulates in the brain of AD patients, bind copper. Aim of this study was to test the hypothesis that the association of Hcy and AD results from a molecular interaction between Hcy and Aβ that is mediated by copper. We established a microtiter plate format thioflavin T aggregation assay to monitor Aβ42 fibrillization. Copper (5 μM) completely prevented Aβ42 (5 μM) fibrillization. Homocysteine in the absence of copper did not impact Aβ42 fibrillization, but physiological concentrations of Hcy (10–100 μM) attenuated the inhibitory effect of copper on Aβ42 fibril formation. These results were qualitatively confirmed by electron microscopy, which did not reveal morphological differences. To compare the toxicity of fibrillar and non-fibrillar Aβ42 exposed to copper or Hcy, rat primary cortical neurons were treated *in vitro* with 5 μM Aβ42 for 72 h. After incubation with 5 μM Aβ42 that had been aggregating in the absence of Hcy or copper, cell viability was reduced to 40%. Incubation with 5 μM Aβ42, in which fibril formation had been prevented or reverted by the addition of 5 μM copper, resulted in cell viability of approximately 25%. Accordingly, viability was reduced to 25% after incubation with 5 μM monomeric, i.e., non-fibrillized, Aβ42. The addition of Hcy plus copper to 5 μM Aβ42 yielded 50% viability. In conclusion, copper prevents and reverts Aβ fibril formation leading rather to formation of lower order oligomers or amorphous aggregates, and Hcy reduces these effects. Such mechanisms may explain the association of hyperhomocysteinemia and AD, leading to novel therapeutic strategies in the prevention and treatment of this disease.

## Introduction

Alzheimer’s disease (AD) is a multifactorial neurodegenerative condition constituting the majority of dementias. Primary feature of AD is neuronal cell loss in the hippocampus and cerebral cortex, areas involved in memory and cognition (Bernardo et al., 2007; Kim et al., 2008). Histopathological characteristics are depositions of amyloid plaques, comprising extracellular accumulations of fibrillar amyloid β (Aβ)-peptide, and the formation of intracellular neurofibrillary tangles composed of hyperphosphorylated tau (P-tau; Hooijmans et al., 2009; Kim and Tsai, 2009). Aβ is produced by cleavage of the amyloid precursor protein (APP) by β-secretase (BACE-1) and γ-secretase, which is comprised of four proteins: presenilin (PS) -1 or -2, PEN, Aph-1 and Nicastrin. Cleavage of APP by β-secretase results in a N-terminal soluble fragment and a C-terminal fragment that is further cleaved by γ-secretase resulting in Aβ peptides. Missense mutations in either APP or PS-1 can cause accumulation of Aβ in hereditary AD. The mechanism leading to Aβ accumulation in the majority of sporadic AD patients is unclear (Mare et al., 2007). Extracellular aggregation of the Aβ-peptide is considered a central and causative phenomenon of AD (Yoshiike et al., 2001; Hooijmans et al., 2009; Zatta et al., 2009; Finder et al., 2010). However, in AD patients, Aβ is also present in elevated amounts within the degenerating neurons, and this may contribute to cell death (Hasegawa et al., 2005).

*In vivo* Aβ has two predominant forms: Aβ1-40 and Aβ1-42 with two additional hydrophobic residues at the carboxyterminus. Aβ1-40 is the main soluble species, whereas Aβ1-42 is the predominant species found in amyloid plaques. The latter is more toxic to neurons and is considered the most amyloidogenic species, most likely responsible for the neuropathology in AD (Hasegawa et al., 2005; Mare et al., 2007; Finder et al., 2010). Amyloid β aggregation is believed to happen in phases: first, Aβ monomers associate into soluble oligomers that then form insoluble oligomers (initial slow nucleation or “seeding”), generating protofibrils, and fibrils (Finder and Glockshuber, 2007; Tõugu et al., 2009).

Whether Aβ forms fibrils *in vitro* in the presence of copper and the nature of these fibrils is currently a subject of debate. Main question is the accelerating or preventing role of copper in amyloid fibril formation, and whether this role is dependent on Cu^2+^ or Aβ concentration and stoichiometry. It has been presented that sub-stoichiometric concentrations of Cu^2+^ accelerate amyloid fibril formation, and supra-stoichiometric concentrations of Cu^2+^ prevent fibrillization (Viles, 2012). There are several studies where Cu^2+^ was reported to inhibit fibril formation and rather form amorphous aggregates (Yoshiike et al., 2001; Raman et al., 2005; Tõugu et al., 2009; Innocenti et al., 2010). On the contrary, the opposing arguments rely mainly on the study of Sarell et al. (2010) where the substoichiometric levels of Cu^2+^ were shown to accelerate fibril formation of Aβ. A recent study of Mold et al. (2013) addresses this dilemma by fluorimetry and transmission electron microscopy (TEM). In this study they show that Cu^2+^, independent of stoichiometry, prevented the formation of ThT-positive amyloid fibrils of Aβ42.

Amyloid plaques are composed of fibrillar Aβ, small amounts of other proteins and transition metals like copper and zinc (Tõugu et al., 2009). Several studies have shown that homeostasis of the transition metals copper and zinc can greatly influence Aβ misfolding and plaque formation. Furthermore, restoring metal ion homeostasis dissolved Aβ plaques in mice and delayed cognitive deficits in AD patients (Zatta et al., 2009). Thus, an interaction between Aβ and copper may be involved in AD pathology (Klevay, 2007a,b).

We have previously shown that homocysteine (Hcy) binds copper, and that this may be an important mechanism of the neurotoxicity of Hcy, as the presence of Hcy can lead to deficiency of copper-dependent enzymes like cytochrome-C-oxidase (White et al., 2001; Apostolova et al., 2003; Linnebank et al., 2006). Hcy is a non-proteinogenic sulfhydryl-containing amino acid formed as an intermediate in the metabolism of methionine (Hasegawa et al., 2005; Bernardo et al., 2007; Kim et al., 2008). Deficiencies of vitamin B12 or folate, common conditions in the elderly, can lead to hyperhomocysteinemia, which is a risk factor for cardio- and cerebrovascular diseases as well as neurodegenerative disorders such as AD (White et al., 2001; Irizarry et al., 2005; Linnebank et al., 2006; Bernardo et al., 2007; Kim et al., 2008). In hyperhomocysteinemic patients, blood copper levels are elevated, possibly due to binding to increased amounts of Hcy (Apostolova et al., 2003; Linnebank et al., 2006). In cell culture, Hcy sensitizes neurons to Aβ toxicity by induction of intraneuronal Aβ accumulation due to speculative mechanisms (Hasegawa et al., 2005). In addition, hyperhomocysteinemia increases Aβ production in rats, probably through enhanced expression of γ-secretase and APP phosphorylation, placing hyperhomocysteinemia upstream of increased Aβ production (Zhang et al., 2009). In this study, we aimed at modelling the interaction between copper, Hcy and Aβ fibril formation.

## Materials and methods

### Origin of reagents

All reagents were ultra pure quality and purchased from Sigma-Aldrich (Buchs, Switzerland) unless otherwise indicated. DL-Homocysteine was minimum 95% titration (Sigma-Aldrich). Recombinant Aβ42 peptide was purchased as a 1,1,1,3,3,3-hexafluoro-2-propanol (HFIP) film, and His6Ala (H6A) mutated recombinant Aβ42 and scrambled recombinant Aβ42 as trifluoroaceticacid (TFA) film from rPeptide (Bogart, Georgia, USA). Solutions were prepared in fresh MilliQ-water.

### Preparation of Aβ42 peptide stocks

To ensure homogenous preparation of the Aβ42 peptide, 1 mg of recombinant peptide HFIP or TFA film was distributed in 50 μg aliquots. All peptides were aliquotted with the same procedure. After addition of 200 μL HFIP to 1 mg peptide, the solution was shortly sonicated, transferred into a Protein LoBind tube (Eppendorf, Hamburg, Germany), and the solvent was evaporated with a constant stream of nitrogen. The peptide film was resuspended in 1 ml of HFIP and, after short vortexing and sonication, dispensed in 50 μg aliquots. HFIP was evaporated under a stream of nitrogen, aliquots were snap-frozen, and stored at −80°C until use as described previously (Wood et al., 1996; Stine et al., 2003).

### Preparation of fresh Aβ42 working solution

For assays, one Aβ42 peptide aliquot was dissolved in 44.4 μl 10 mM NaOH, pH 12, to yield a stock solution of approximately 250 μM. The aliquot was vortexed, sonicated, vortexed again shortly, spun down and placed on ice until use. The resuspension of the Aβ1-42 film in 10 mM sodium hydroxide was adapted from Teplow (2006). The low NaOH concentration reassured the rapid pH neutralization to 7.4 upon dilution into the experimental buffer (Teplow, 2006).

The concentration of the Aβ42 solution was determined via absorbance at *λ* = 280 nm measured with NanoDrop UV/Vis spectrophotometer (NanoDrop Technologies, Wilmington Delaware, USA). Concentration was calculated using a molar extinction coefficient of *ɛ* = 1730 M^−1^ cm^−1^ (Finder et al., 2010).

### Thioflavin T aggregation assays

To study amyloid fibril formation, 5 μM Aβ42 peptide was mixed with 50 μM Thioflavin T (ThT) in 10 mM sodium phosphate solution, 500 mM NaCl, and 0.1 mM HCl to a final volume of 100 μl. Different concentrations of ZnCl_2_, CuCl_2_ and Hcy were added to selected samples after 0 or 120 min of measurement, respectively. Samples were incubated in a flat bottom microtiter plate, and the increase in ThT fluorescence was measured via top-beam irradiation (*λ*_Ex_ = 450 nm, *λ*_Em_ = 510 nm) with a lamp energy of 5000 (arbitrary unit) and a counting time of 0.1 s by Berthold Mithras LB 940 (Berthold Technologies GmbH, Regensdorf, Switzerland). Values were recorded every 2 min with constant orbital shaking at slow speed between the measurements. Temperature was controlled to 30°C.

### Cytotoxicity

Rat primary cortical neuron cultures were prepared as described (Finder et al., 2010). Neurons were plated in Neurobasal media (GIBCO, Invitrogen, Basel, Switzerland) with B-27 supplement (GIBCO) and L-glutamine (GIBCO) on poly-L-ornithine pre-coated 96-well plates at a density of approximately 10,000 cells per well. Cultures were maintained in a humidified 7% CO_2_ incubator. Primary cortical neuron cultures were treated with Aβ-fibrils on day 6 *in vitro*. For cytotoxicity assessment, aggregation reactions were performed with 10 times higher concentrations and in the absence of ThT. Thioflavin T does not affect aggregation kinetics, but influences cytotoxicity measurements (Finder et al., 2010). Otherwise reaction parameters were as described above.

After reaching aggregation plateau (after 3 h), fibril suspensions were transferred in 1:10 (v/v) ratio in relation to cell culture medium in the wells. Final concentrations on the cells were: 5 μM Aβ, 5 μM CuCl_2_ and 50 μM Hcy. Reaction mixture without Aβ, CuCl_2_ or Hcy was used as negative control. Just before treatment with diluted fibrils, half of the culture medium on cells was aspirated and replaced with fresh Neurobasal medium. Assays were performed minimum as triplicates. After 72 h cell viability was quantified by a colorimetric 3-(4,5-Dimethylthiazol-2-yl)-2,5-diphenyltetrazolium bromide (MTT; Keskitalo et al., 2007). Briefly, treatment medium was removed from cells and replaced by Neurobasal medium with MTT. After incubation for 2 h, lysis buffer (10% SDS, 10 mM HCl) was added for the incubation of cell cultures at 37°C overnight. Absorbance was measured on the next day (Berthold Mithras LB 940, Berthold Technologies GmbH), and relative survival to control (reaction mixture without Aβ, CuCl_2_ or Hcy) was calculated.

### Cell morphology by immunofluorescent staining

Approximately 100,000 rat primary cortical neurons were seeded in 24-well plates onto glass coverslips pre-coated with poly-L-ornithine in water. On *in vitro* day 6, a 24 h-incubation with the 1:10 diluted fibrils was started. As in the MTT-assay, aggregation reactions were performed with 10 times higher reaction concentration and without ThT, half of the culture medium on cells was aspirated just before adding the aggregates. Due to the aggregation assay results and the physiologically occurring Hcy levels, we chose samples incubated with 5 μM Aβ, 5 μM CuCl_2_ and 50 μM Hcy to be shown in the results. Cells were stained minimum as duplicates. 5 μM non-fibrillar Aβ42 was used as a control.

After incubation with the fibrils the cells were fixed for 15 min at room temperature with 4% paraformaldehyde in PBS. The coverslips were rinsed with PBS, and washed three times with 0.05% Triton X-100 in TBS for 10 min each. After blocking with 5% goat serum (Millipore, Zug, Switzerland), 5% horse serum (GIBCO) and 0.2% Triton X-100 in TBS for 60 min, the coverslips were incubated with primary antibodies in blocking buffer (anti-MAP2 1:1500, Synaptic Systems, Germany; 1:100 anti-human APP 6E10, Covance, Princeton, New Jersey, USA) in a humidified chamber overnight at 4°C. On the following day coverslips were washed three times with 0.05% Triton X-100 in TBS for 10 min each, blocked for 30 min at room temperature in blocking solution, and incubated with 1:300 diluted secondary antibodies (anti-rabbit Alexa488 (Invitrogen) and anti-mouse Cy3 (Jackson ImmunoResearch, West Grove, Pennsylvania, USA)) in blocking buffer for 2 h at room temperature. After washing, cell nuclei were stained with 2-(4-amidinophenyl)-1H-indole-6-carboxamidine (DAPI), and coverslips were mounted on glass slides with Hydromount (Chemie Brunschwig, Basel, Switzerland). Staining was examined using a Zeiss ImagerZ1 microscope (Zeiss, Oberkochen, Germany). All images were taken with a 20× objective.

### Negative-stain electron microscopy of aggregates

Aliquots of the aggregation reactions without ThT were analyzed, when plateau was readily reached after 4 h agitation at 30°C. 3 μl of each sample was adsorbed to 300 mesh carbon-coated copper grids for 1 min and stained with 2% uranyl acetate in water for 15 s three times. After staining grids were washed with water and allowed to dry before TEM. TEM was performed on a Philips CM 12 microscope at 100 MeV.

### Statistics

Statistical analysis was run using IBM SPSS Statistics 20 (IBM, Armonk, New York, USA), and significance was calculated using one-way ANOVA with Bonferroni’s *post hoc* test for multiple comparisons. Statistical significance was considered as *p* < 0.05.

All experiments were repeated three times with *n* ≥ 3 samples for each experimental condition.

## Results

### Homocysteine and Aβ42 compete for CuCl_2_, but not for ZnCl_2_, in ThT aggregation assay

**Figure 1:**
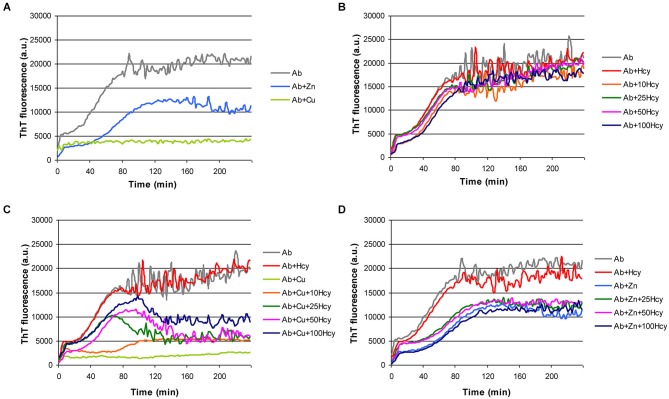
**Effect of metal ions and homocysteine (Hcy) on Aβ42 fibrillization as observed in ThT-assay**. Fibrillization of 5 μM Aβ42 (light gray) in the presence of **(A)** 5 μM ZnCl_2_ (light blue) or 5 μM CuCl_2_ (light green), **(B)** increasing concentrations of Hcy (5 μM—red, 10 μM—orange, 25 μM—dark green, 50 μM—pink, and 100 μM—dark blue), **(C)** 5 μM Hcy (red) or 5 μM CuCl_2_ (light green), or 5 μM CuCl_2_ together with increasing Hcy concentration (10 μM—orange, 25 μM—dark green, 50 μM—pink, and 100 μM—dark blue), and **(D)** 5 μM Hcy (red) or 5 μM ZnCl_2_ (light blue), or 5 μM ZnCl_2_ together with increasing Hcy concentration (25 μM—dark green, 50 μM—pink, and 100 μM—dark blue). All components were added to the reaction mixture directly at the beginning of the fibrillization reaction.

We established a microtiter plate format ThT aggregation assay for fast and reproducible monitoring of Aβ42 fibrillization in the presence of Hcy and the transition metals copper and zinc. As previously reported, copper and zinc inhibited the formation of ThT reactive beta-sheet structures of Aβ (Yoshiike et al., 2001; House et al., 2004). In our experiments ZnCl_2_ reduced Aβ42 fibrillization by extending the lag phase, slightly decreasing the slope, and diminishing the final plateau (Figure 1A). CuCl_2_ (5 μM) completely prevented Aβ42 (5 μM) fibrillization (Figure 1A). Homocysteine alone at increasing concentrations had no effect on Aβ fibril formation (Figure 1B), but concentration-dependently reduced the inhibitory effect of CuCl_2_ on Aβ42 fibrillization (Figure 1C). No such interaction on Aβ42 aggregation was observed between Hcy and ZnCl_2_ (Figure 1D).

### Homocysteine does not alter Aβ42 fibril morphology

**Figure 2:**
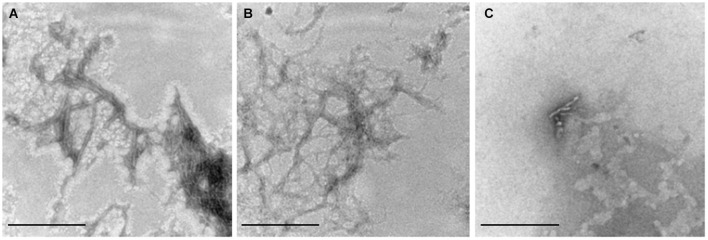
**Visualization of Aβ42 fibrils**. 110,000x transmission electron microscopy images of the end point products of Aβ42 fibrillization after 4 h of aggregation at 30°C. **(A)** 5 μM Aβ42 alone, **(B)** 5 μM Aβ42 + 50 μM Hcy and **(C)** 5 μM Aβ42 + 5 μM CuCl_2_. Aβ42, Hcy and CuCl_2_ were all added at the beginning of the ThT-assay. Scale bar represents 100 nm.

To decide whether Hcy or copper have qualitative effects on Aβ aggregation, we analyzed the morphology of the aggregates of 5 μM Aβ42, 5 μM Aβ42 plus 50 μM Hcy and 5 μM Aβ42 plus 5 μM CuCl_2_ by TEM after 4 h aggregation (Figure 2). TEM images confirmed the observations from ThT aggregation assays that Hcy alone does not alter Aβ42 fibrillization, as the Aβ42 fibrils formed in the presence of Hcy were alike to fibrils formed without Hcy showing a high number of mature fibrils (Figures 2A,B). In the presence of CuCl_2_, only few aggregates were found with decreased fibril length and complexity (Figure 2C).

### Toxicity of Aβ42 fibrils is increased in the presence of CuCl_2_ and decreased in the presence of homocysteine or homocysteine plus CuCl_2_

**Figure 3:**
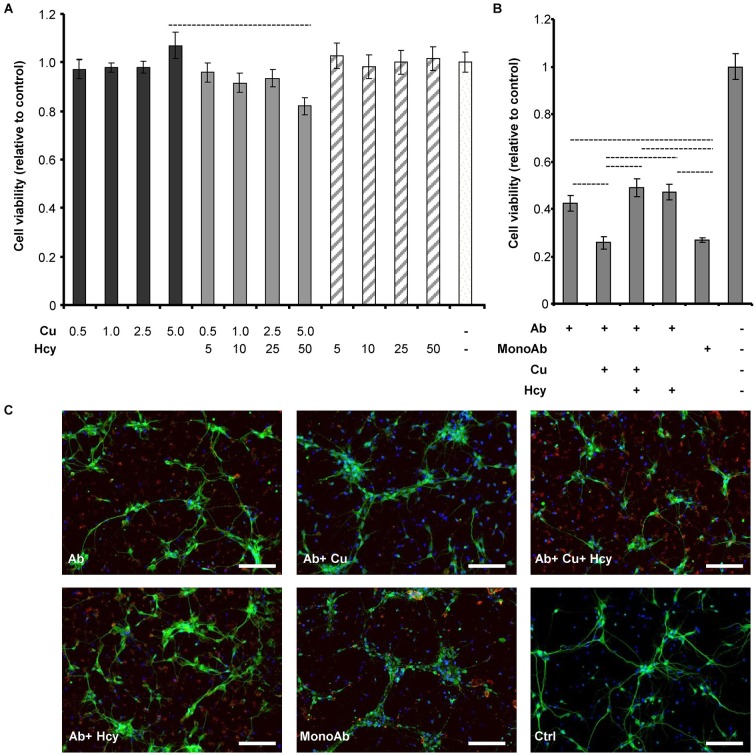
**Cell viability and morphology of rat primary cortical neurons after treatment with CuCl_2_ and Hcy in the absence or presence of Aβ42**. Aggregations for viability and morphology studies were performed identically to previous aggregation assays, but lacking ThT due to its cytotoxicity. After 4 h agitation, samples were subsequently collected for the assessments. **(A)** Primary neurons were incubated for 72 h with increasing μM concentrations of CuCl_2_ (dark gray), CuCl_2_ + Hcy (light gray), or Hcy (stripes) without Aβ42, to study their individual cytotoxicity. Control sample (aggregation assay reaction mixture) is visualized in the white column. **(B)** Effect of CuCl_2_ and Hcy on Aβ42-induced toxicity. 5 μM Aβ42; 5 μM Aβ42 + 5 μM CuCl_2_; 5 μM Aβ42 + 5 μM CuCl_2_ + 50 μM Hcy; 5 μM Aβ42 + 50 μM Hcy were incubated on the cells for 72 h. As controls non-fibrillar 5 μM Aβ42 (MonoAb) and aggregation assay reaction mixture without Aβ42 were used. All values are relative to reaction mixture control sample ± S.D. **(C)** Immunofluorescent staining of primary cortical neurons after 24 h incubation. Antibody against neuronal marker, MAP2 (green), visualizes the changes of neuronal morphology; whereas anti-human APP (red) shows the Aβ aggregates and DAPI (blue) the cell nuclei. Concentrations were as indicated in **(B)**. Scale bar represents 100 μm.

To be able to conclude whether cytotoxicity of mixtures of copper, Hcy and Aβ is caused by changes in the fibrillar status of Aβ42, we examined the cytotoxic effects of CuCl_2_, Hcy and the two together without Aβ42 (Figure 3A). Rat primary cortical neurons were treated on day 6 *in vitro* for 72 h with increasing concentrations of CuCl_2_ (0.5–5.0 μM), Hcy (5–50 μM) or CuCl_2_ plus Hcy in incubation mixtures previously incubated for 4 h at 30°C. No significant toxicity was observed in neurons treated with the selected concentrations of CuCl_2_ or Hcy alone. In line with previous results, the co-incubation of Hcy plus CuCl_2_, i.e., with homocysteine/copper-complexes, showed a concentration-dependent toxicity (White et al., 2001; Linnebank et al., 2006).

Next we studied the toxicity of Aβ42 fibrils formed in the presence of CuCl_2_, Hcy or both (Figure 3B). Viability of primary neurons decreased to 40% after 72 h incubation with 5 μM Aβ42 forming fibrils alone. Additional presence of 50 μM Hcy or 5 μM CuCl_2_ plus 50 μM Hcy resulted in a slightly higher viability of 50%. 5 μM Aβ42 incubated in the presence of 5 μM CuCl_2_ showed a significant increase in toxicity reducing cell viability to 25%. The same viability was observed after incubation with 5 μM monomeric Aβ. This shows that most likely CuCl_2_ induces the formation of lower order oligomers or amorphous aggregates of Aβ that have high cytotoxicity, whereas Hcy diminishes this effect and does not obviously contribute to cytotoxicity itself under the selected experimental conditions.

Observations from cytotoxicity studies were confirmed by morphological analysis of neurons treated with Aβ42 aggregates collected after 4 h of aggregation without ThT (Figure 3C). Cells incubated with 5 μM Aβ42 plus 5 μM CuCl_2_ were shrunken and presented fewer neurites, resembling the morphology of cells treated with 5 μM monomeric Aβ42. In both, Aβ42 plus CuCl_2_ and monomeric Aβ42 treated cells, also anti-APP staining patterns were similar visualizing fewer and smaller amyloid plaque-like structures. Aβ42-stainings in other treatment conditions were similar to each other. Vehicle (without Aβ42) serving as a negative control for anti-APP staining showed no such effects.

### CuCl_2_ has limited effects on the aggregation of H6A mutated Aβ42

**Figure 4:**
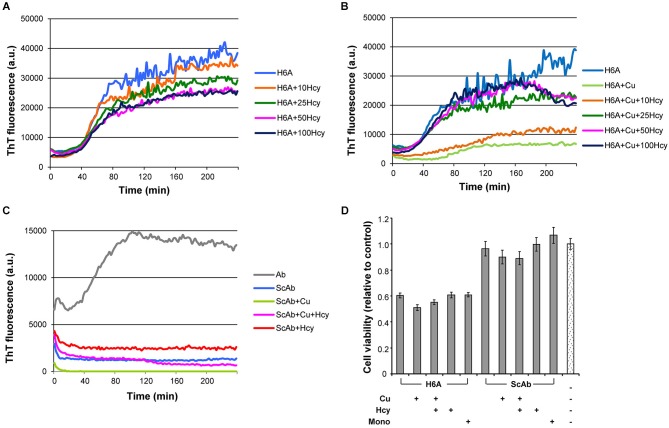
**Fibrillization of H6A mutated Aβ42 and scrambled Aβ42 in ThT-aggregation assay, and their cytotoxicity in rat primary cortical neurons**. Fibrillization of 5 μM H6A mutated Aβ42 (H6A; light blue) **(A)** in the presence of increasing concentrations of Hcy (10 μM—orange, 25 μM—dark green, 50 μM—pink, and 100 μM—dark blue), **(B)** with 5 μM CuCl_2_ and increasing concentrations of Hcy (10 μM—orange, 25 μM—dark green, 50 μM—pink, and 100 μM—dark blue). **(C)** 5 μM scrambled Aβ42 (ScAβ; light blue) does not form fibrils when incubated alone or together with 5 μM CuCl_2_ or 5 μM CuCl_2_ + 50 μM Hcy or 50 μM Hcy. **(D)** Cell viability of rat primary neurons after 72 h incubation with H6A fibrils or ScAβ incubated under same conditions. Samples from aggregation assay without ThT, but with CuCl_2_, Hcy or both, were collected at the plateau after 4 h incubation. Concentrations were 5 μM H6A or ScAβ; 5 μM H6A or ScAβ + 5 μM CuCl_2_; 5 μM H6A or ScAβ + 5 μM CuCl_2_ + 50 μM Hcy; 5 μM H6A or ScAβ + 50 μM Hcy. As a control non-fibrillar 5 μM H6A or ScAβ (Mono) and aggregation assay reaction mixture without H6A or ScAβ were used. All values are relative to reaction mixture control sample ± S.D.

Fibrillization of His6Ala-mutated Aβ42 (H6A), which has low affinity to copper (Sacco et al., 2012), was studied to examine the specificity of copper induced inhibition of Aβ42 aggregation. Homocysteine only caused a minor concentration-dependent reduction in the maximum of ThT fluorescence in H6A fibrillization, which might have been due to unspecific variation in the ThT signal (Figure 4A). At Hcy concentrations higher than 50 μM, aggregation curves remained unchanged. Addition of 5 μM CuCl_2_ to the aggregation reaction containing 5 μM H6A inhibited its fibrillization leading to a longer lag phase, a decreased slope and plateau. Nonetheless, CuCl_2_ was not able to completely prevent H6A fibrillization (Figure 4B). The effect of CuCl_2_ on H6A was thus decreased compared to its effects on wild-type peptides. Increasing concentrations of Hcy (10–100 μM) in the aggregation reaction of 5 μM H6A plus 5 μM CuCl_2_ reversed the inhibitory effect of CuCl_2_ on H6A fibrillization (Figure 4B). The concentration of Hcy needed to restore H6A fibril formation was smaller than in the case of wild-type Aβ42. As H6A has a lower affinity to copper than Aβ42, this underscores that there is a competition in binding of copper between Aβ42 and Hcy as underlying mechanism of the interaction of Hcy and copper on Aβ fibrillization. The increase in ThT fluorescence is specific to the formation of cross-pleated β-sheets. Accordingly, ThT fluorescence of incubation mixtures containing scrambled Aβ42 (ScAβ), a peptide derivative of Aβ42 that does not form fibrils, did not differ after addition of either 5 μM CuCl_2_, or 50 μM Hcy or 5 μM CuCl_2_ plus 50 μM Hcy confirming that the above described results did not result from artefacts induced by Hcy or copper in the incubation mixtures (Figure 4C).

### Toxicity of Aβ42, H6A and ScAβ to primary cortical neurons

In primary neuron cultures, fibrils of 5 μM H6A, formed either in the absence or presence of 5 μM CuCl_2_, 50 μM Hcy, or a combination of both, each reduced cell viability to approximately 60% after 72 h incubation (Figure 4D). Viability of neurons treated with 5 μM ScAβ, incubated in the absence or presence of 5 μM CuCl_2_, 50 μM Hcy or both, remained at approximately 90%. The overall toxicity of the H6A was significantly higher than the one of ScAβ, but lower than of Aβ42.

### Addition of CuCl_2_ to already aggregated samples untangled Aβ42 fibrils

To study whether copper supplementation could be used to revert aggregation, we performed a simple aggregation assay in which CuCl_2_ was added to an already fibrillized sample of either Aβ42 alone or of Aβ42 incubated in the presence of 15 μM or 50 μM Hcy. We selected these Hcy concentrations as they define the lower range of Hcy plasma concentrations in mild and intermediate hyperhomocysteinemia, respectively (Stanger et al., 2009).

**Figure 5:**
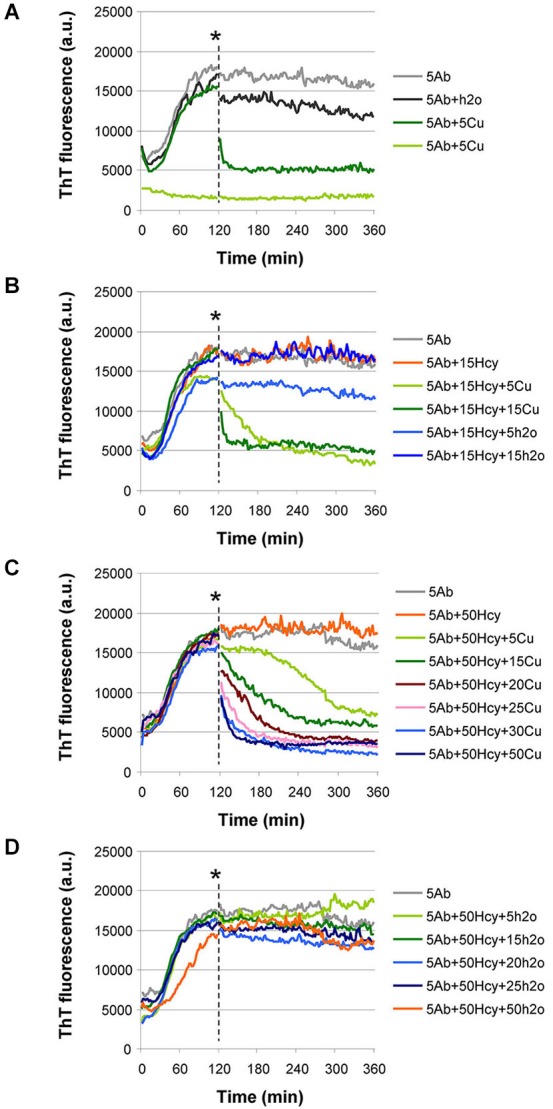
**Aβ42 fibrils untangle by addition of CuCl_2_ at timepoint 120 min in ThT aggregation assay. (A–C)** CuCl_2_ was added at the point indicated by an asterisk. **(A)** Addition of 5 μM CuCl_2_ caused a drastic reduction in ThT fluorescence when added to an aggregation reaction of 5 μM Aβ42 (dark green). Water, in equal volume, (H_2_O, dark gray) did not induce reduction in ThT fluorescence. As controls, aggregation of 5 μM Aβ42 (light gray) and 5 μM Aβ42 + 5 μM CuCl_2_, added at the beginning of the reaction (light green), are shown. **(B)** When Aβ42 fibrils are formed in the presence of 15 μM Hcy (orange), their untangling after CuCl_2_ addition depends on the molar ratio between CuCl_2_ and Hcy (5 μM CuCl_2_—light green and 15 μM CuCl_2_—dark green). Water added in equal volumes to CuCl_2_ did not change ThT fluorescence (light and dark blue). As a control aggregation of 5 μM Aβ42 (light gray) is shown. **(C)** After CuCl_2_ addition Aβ42 fibrils that were previously formed in high Hcy concentration (5 μM Aβ42+ 50 μM Hcy) untangled. Degree of untangling was dependent on the molar ratio of Aβ42 to added CuCl_2_ and on the molar ratio of Hcy to added CuCl_2_ concentration (5 CuCl—light green, 15 μM CuCl_2_—dark green, 20 μM CuCl_2_—purple, 25 μM CuCl_2_—light pink, 30 μM CuCl_2_—light blue, and 50 μM CuCl_2_—dark blue). **(D)** Water addition at timepoint 120 min did not induce changes in the ThT fluorescence of the reactions of 5 μM Aβ42 + 50 μM Hcy. Water was added in equal volumes as CuCl_2_ in panel **(C)**.

The addition of 5 μM CuCl_2_ to a sample of 5 μM Aβ42 at time point 120 min drastically reduced ThT fluorescence, indicating untangling of Aβ42 fibrils (Figure 5A) similar to the preventive effect of copper on fibrillization (Figure 2C). Only a minor reduction in ThT fluorescence was observed after addition of an equal volume of water (vehicle in which copper had been dissolved) to already fibrillized 5 μM Aβ42 at the same time point as negative control (Figures 5A,B). After addition of increasing CuCl_2_ concentrations (5–15 μM), Aβ42 fibrils that had formed in the presence of 15 μM or 50 μM Hcy also untangled (Figures 5B,C). The decrease in ThT fluorescence depended on the ratio between copper and Hcy (Figures 5B,C). ThT fluorescence remained unchanged after vehicle additions (Figure 5D).

## Discussion

Hyperhomocysteinemia is a risk factor for AD, in which Aβ fibrillization plays an important role. Our study suggests that copper is a link between Hcy and Aβ. First, via a ThT assay and TEM, we confirmed that *in vitro* CuCl_2_ prevents and reverts Aβ fibril formation (House et al., 2004; Bolognin et al., 2011; Chen et al., 2011; Figures 1A–C, 5A–C). Addition of CuCl_2_ results in decreased Aβ fibril length and complexity i.e., lack of higher order aggregates (Figure 2C). The underlying mechanism is most likely connected to the ability of CuCl_2_ to prevent the formation of Aβ42 beta-sheets* in vitro* (Yoshiike et al., 2001; House et al., 2004), but due to the nature of ThT assay we cannot completely rule out the possibility of the formation of oligomers or amorphous aggregates. However, Hcy did not affect the fibrillar structure of Aβ as seen in ThT assay and in TEM images (Figures 1B, 2B).

Our ThT aggregation assay results of CuCl_2_ and Aβ are in-line with other published results with similar experimental setup. It has been presented that in experimental conditions where fibril formation is fast, metal ions lower concentration of free peptide and thus inhibit fibrillization (Tõugu et al., 2009). In conditions with slow fibril formation metal ions enhance fibril formation by metal-induced aggregates that can turn into fibrils (Sarell et al., 2010). Differences in experimental setup are also greatly influencing the outcome of fibrillization studies. This is summarized in a recent publication by Viles (2012) and shows multiple differences associated with changes in stoichiometry, used peptide preparation, concentration and study technique.

Cytotoxicity experiments performed in the absence of Aβ42 showed that CuCl_2_ or Hcy alone had no effect on cell viability (Figure 3A). In accordance to previous studies, cytotoxicity was elevated when cells were co-incubated with CuCl_2_ and Hcy, confirming the toxicity of Hcy-copper-complexes (Figure 3A; White et al., 2001). In accordance with our ThT assay data and TEM images, toxicity of mature Aβ42 fibrils to primary neurons was high, and Aβ fibrils formed in the presence of Hcy and CuCl_2_ showed similar toxicity (Figures 1C, 2A–C, 3B,C). Aβ42 co-incubated in 1:1 molar ratio with CuCl_2_ showed the same level of toxicity as monomeric Aβ42 underlining that CuCl_2_ effectively inhibited the formation of bigger, more mature fibrils during ThT aggregation assays (Figures 2C, 3B; House et al., 2004; Chen et al., 2011). Is has been reported that the fibrillar status of Aβ42 affects its cytotoxicity in cultured neurons: small soluble Aβ42 dimers and oligomers cause higher toxicity than bigger fibrillar forms (Klyubin et al., 2005; Lesné et al., 2006; Agnati et al., 2007; Ferreira et al., 2007; Haass and Selkoe, 2007; Ono et al., 2009).

As a certain time-span is required for Aβ preparations in contact with primary neurons to exert the cytotoxic properties, we cannot completely rule out that during the 72 h incubation a modification of the Aβ species can occur. However, as a result of our Aβ ThT aggregation assays we observed that the addition of CuCl_2_ almost completely prevented the formation of higher order aggregates (sensitive to ThT fluorescence). As CuCl_2_ itself did not have a measurable influence on cell viability, we infer that the observed cytotoxicity is due to Aβ monomers and potentially aggregates of lower molecular weight, such as Aβ di- and oligomers, Aβ-derived diffusible ligands or protofibrils (all probably being ThT fluorescence negative) that may have formed during the incubation period.

Copper has four coordinating ligands in human Aβ: 3N and 1O, which involve His6, His13, His14 and possibly Tyr10, carboxylate group of Asp1, the amide of Ala2, and the N-terminal amine. To date, no consensus of exact coordinating ligands exists. When single histidines were mutated to alanine, Hong et al. (2010) observed that His6 has three times higher copper binding constant than His13 or His14. His6 also requires less conformational changes upon copper binding, making it more entropy-favored. His6 was concluded to be ubiquitously involved in copper binding, accounting for 50% of the Aβ bound Cu(II). In our experiments, Hcy had only minor effect on the fibril formation of H6A mutant of Aβ42 with reduced affinity to copper (Figure 4A). Also the effect of CuCl_2_ was weaker in H6A fibrillization, and the concentration of Hcy needed to restore H6A fibril formation was reduced compared to wild type Aβ42 (Figures 4B, 1C). This allows the speculation that the amount of mature fibrils i.e., higher order aggregates in this sample was higher compared to the wild type Aβ42. In accordance to this, H6A fibrils also showed less pronounced cytotoxicity in the presence of CuCl_2_ (Figure 4D). The control, ScAβ, expectedly showed no fibrils in ThT-assay (Figure 4C). Accordingly, ScAβ was not cytotoxic and this was not influenced by incubation with CuCl_2_ or CuCl_2_ plus Hcy (Figure 4D). In ScAβ the copper binding sites are lost, confirming that binding of copper to Aβ was the decisive mechanism for the observed differences in the ThT assay and the cytotoxicity experiments of the different combinations.

The addition of CuCl_2_ to already fibrillized Aβ led to untangling of fibrils (Figures 5A–C). This does not necessarily mean that copper is relevant for Aβ fibrillization *in vivo*, however, mice with defective copper transport have decreased brain copper levels together with increased amounts of amyloid plaques. When these mice are crossed with Wilson’s disease mouse model, the offspring have increased brain copper levels, less amyloid plaques and a longer life span (Phinney et al., 2003). When copper sulphate was added to the drinking water of mice susceptible to amyloid accumulation, less accumulation was observed (Bayer et al., 2003). In patients with mild to moderate AD, plasma copper negatively correlates with cognitive abilities (Pajonk et al., 2005; Kessler et al., 2006). In addition, AD patients show elevated serum levels of free copper (serum copper not bound to ceruloplasmin) (Squitti et al., 2004, 2005, 2006, 2009, 2011; Capo et al., 2008) while autopsy samples of hippocampus and amygdala from AD patients showed generally reduced copper contents (Deibel et al., 1996; Klevay, 2008).

In contrast, in the amyloid plaques, copper concentration can be as high as 400 μM, although normal brain extracellular concentration is 0.2–1.7 μM (Gutteridge, 1984; Kardos et al., 1989; Linder and Hazegh-Azam, 1996; Lovell et al., 1998; Schümann et al., 2002; White et al., 2004; Squitti et al., 2006). This data seems to be in contrast to our observation that CuCl_2_ prevents amyloid fibril formation. However, in our experiments, CuCl_2_ alone prevented and reverted aggregation, whereas Hcy plus CuCl_2_ did not. Thus, it would be interesting to analyze whether copper in amyloid plaques of AD patients is bound to Hcy or other molecules. Moreover, the inhibitory effect of CuCl_2_ on fibrillization was concentration-dependent (Figures 5B,C). Thus, one may speculate that in the copper-rich plaques of AD patients, copper levels may not have reached the necessary concentration.

Serum Hcy concentrations over 14 μM are an independent risk factor for the development of AD (Seshadri et al., 2002). In our study, the addition of Hcy alone did not change Aβ42 fibril formation (Figure 1B), although the addition of Hcy slightly reduced Aβ42 toxicity *in vitro* (Figure 3B). In co-incubation experiments, Hcy concentration-dependently reduced the inhibitory effects of CuCl_2_ on Aβ42 fibrillization suggesting that Hcy and Aβ compete for copper binding, i.e., homocysteine-bound copper has reduced or no effects on Aβ42 fibril formation (Figure 1C). Two different complexes are possible between Hcy and copper, showing molar ratios of 1:1 or ≤1:3 (Apostolova et al., 2003). Similarly, the majority of the copper-Aβ complexes form with a 1:1 stoichiometry at physiological pH (Karr et al., 2005; Syme and Viles, 2006; Tõugu et al., 2008; Faller and Hureau, 2009). For H6A, Hcy was more effective in neutralizing the effect of CuCl_2_ on fibril formation confirming that CuCl_2_ is less effective in preventing fibrillization of this mutated peptide due to its reduced affinity to copper (Figure 4B).

In summary, this study shows that both Hcy and Aβ42 bind and compete for copper. Copper prevents and reverts fibril formation by binding to Aβ42 and thereby increases Aβ toxicity. Homocysteine builds toxic complexes with copper and concentration-dependently prevents the effects of copper on Aβ42 fibrillization. In the presence of Aβ42, neurotoxicity of copper is reduced giving rise to the speculation that one physiological Aβ function might be the prevention of copper neurotoxicity. Due to complex building, hyperhomocysteinemia reduces the availability of free copper, which in the light of our results, likely increases amyloid plaque formation. Acute presence of high copper concentrations untangle aggregates leading to high concentrations of mono- or oligomeric Aβ42-copper complexes causing marked neurotoxicity. The analysis of the interaction between Aβ42, copper and Hcy in patients may lead to novel therapeutic strategies in the prevention and treatment of AD.

## Conflict of interest statement

The authors declare that the research was conducted in the absence of any commercial or financial relationships that could be construed as a potential conflict of interest.
